# Analysis of risk factors and development of a prediction model for long-term prognosis in patients with ischemic heart failure after percutaneous coronary intervention

**DOI:** 10.3389/fcvm.2025.1545079

**Published:** 2025-10-27

**Authors:** Lifang Su, Xianghua Fu, Yunfa Jiang, Yanbo Wang, Boyan Tian, Yang Fu, Qing Wang, Wei Zhi, Yi Li, Zhengkun Guan, Xinshun Gu

**Affiliations:** ^1^ Department of Cardiology, The Second Hospital of Hebei Medical University, Shijiazhuang, China; ^2^ Department of Cardiac Ultrasound, The Second Hospital of Hebei Medical University, Shijiazhuang, China; ^3^ Department of Cardiology, The Fourth Hospital of Hebei Medical University, Shijiazhuang, China

**Keywords:** ischemic heart failure, percutaneous coronary intervention, major adverse cardiac events, risk factors, prediction model

## Abstract

**Background:**

This study aimed to investigate the factors influencing the long-term prognosis of patients with ischemic heart failure (IHF) after percutaneous coronary intervention (PCI) and to develop and validate a nomogram prediction model based on these key factors.

**Methods:**

In this single-center and retrospective study, consecutive patients diagnosed with IHF who underwent PCI at the main campus of the Second Hospital of Hebei Medical University between January 2019 and September 2023 were included. A nomogram prediction model was developed based on key factors identified by Cox regression and least absolute shrinkage and selection operator (LASSO) regression. In addition, the patients treated at the branch campus of the Second Hospital of Hebei Medical University during the same period were included for external validation.

**Results:**

The factors significantly associated with major adverse cardiovascular event (MACE) included age, New York Heart Association (NYHA) classification III or IV, residual diseased coronary arteries ≥2, left ventricular ejection fraction (LVEF), left ventricular end-diastolic dimension (LVEDD), and the application of angiotensin receptor–neprilysin inhibitor (ARNI) during follow-up. The nomogram prediction model based on these six factors had an area under the curve (AUC) of 0.764 (95% CI: 0.680–0.847) for the 5-year receiver operating characteristic (ROC) analysis, and the model's concordance index (C-index) was 0.713, indicating good discriminative ability at the 5-year mark. Calibration curve and decision curve analysis demonstrated the model's consistency and clinical utility. The external validation of the model yielded an AUC of 0.707, and the C-index was 0.691. Multivariate Cox regression showed that NYHA classification III or IV, residual diseased coronary arteries ≥2, and LVEDD were independent risk factors for MACE, while the use of ARNI during follow-up was an independent protective factor.

**Conclusions:**

The nomogram prediction model, incorporating age, NYHA classification III or IV, residual diseased coronary arteries ≥2, LVEF, LVEDD, and the use of ARNI during follow-up, demonstrated strong predictive value for long-term MACE in patients with IHF after PCI. NYHA classification III or IV, residual diseased coronary arteries ≥2, and LVEDD were identified as independent risk factors for MACE, while the use of ARNI during follow-up was found to be a protective factor.

## 1 Introduction

Coronary artery disease (CAD) is the leading cause of heart failure (HF) (1, 2). Ischemic heart failure (IHF) is characterized by severe left ventricular systolic dysfunction resulting from extensive CAD. For patients with IHF, revascularization—primarily through percutaneous coronary intervention (PCI) or coronary artery bypass grafting (CABG)—is recommended in conjunction with optimal medical therapy (OMT). Most studies have demonstrated that revascularization can improve cardiac function and clinical outcomes in patients with IHF compared with OMT alone (3–5). For IHF patients, particularly those with multivessel CAD, current guidelines recommend CABG as the first-line treatment, provided they are suitable for surgery (6, 7). However, not all patients will benefit equally from CABG; considerations such as coronary artery anatomy, comorbidities, and surgical risk need to be taken into account (8, 9).

PCI remains an important revascularization strategy, although its benefits in patients with IHF are still debated. Some studies have suggested that PCI offers outcomes comparable to CABG in terms of long-term prognosis in patients with CAD and a left ventricular ejection fraction (LVEF) of ≤35% (10). However, the REVIVED study showed that PCI does not reduce the incidence of all-cause death or hospitalization for patients with ischemic ventricular dysfunction (11). At present, most studies focus on the different effects of PCI and CABG on the long-term prognosis of patients with IHF, and few studies focus on the risk factors affecting different outcomes, especially on the factors affecting the long-term prognosis of patients with IHF after PCI. Moreover, prediction models can combine multiple influencing factors to enhance the accuracy of prognosis prediction. To date, no simple and effective prediction model exists to assist clinicians in evaluating the long-term prognosis of IHF patients following PCI. Therefore, this study aimed to investigate the factors influencing the long-term prognosis of patients with IHF after PCI and to construct and evaluate a prediction model based on these factors, which will help clinicians more effectively identify high-risk patients and implement timely interventions.

## 2 Materials and methods

### 2.1 Study design and patient population

This single-center retrospective study included consecutive patients with CAD combined with HF who underwent PCI at the main campus of the Second Hospital of Hebei Medical University between January 2019 and September 2023. In addition, the patients treated at the branch campus of the Second Hospital of Hebei Medical University during the same period were included for external validation. All the patients must meet the same inclusion criteria.

This study was approved by the Research Ethics Committee of the Second Hospital of Hebei Medical University.

The inclusion criteria were as follows: (1) patients over 18 years old and (2) those with coronary angiography showing ≥70% stenosis in the main coronary artery and its major branches, with or without stenosis of the left main artery ≥50% who underwent PCI during hospitalization. The main coronary territories considered in this study were the left anterior descending artery (LAD), the left circumflex coronary artery (LCX), and the right coronary artery (RCA). (3) Patients whose echocardiography at admission indicated regional wall motion abnormalities with LVEF <50% were also included in the study.

The exclusion criteria were as follows: (1) patients with primary myocardiopathy, congenital heart disease, rheumatic heart disease, or other non-ischemic myocardial diseases; (2) patients who had experienced acute myocardial infarction within one month; (3) patients undergoing emergency surgery or hemodynamic instability (e.g., preoperative shock and cardiopulmonary resuscitation); (4) patients with a concomitant valve procedure or other cardiac surgeries; (5) patients with any form of tumor compromising long-term survival; and (6) patients with incomplete clinical data or lost to follow-up.

All patients were divided into the major adverse cardiovascular event (MACE) group and the non-MACE group based on the occurrence of MACE during the follow-up.

### 2.2 Data collection

Baseline clinical characteristics were collected for all patients, including demographic data, medical history, prescribed medications, laboratory results, echocardiographic parameters, and coronary interventional details. Follow-up data were obtained via outpatient visits or telephone contact.

All patients received standard antiplatelet therapy (aspirin and an oral P2Y12 inhibitor) for 1 year, followed by aspirin monotherapy.

Echocardiography was performed independently by two experienced technicians using standard techniques based on the American Society of Echocardiography and the European Association of Cardiovascular Imaging.

The coronary angiography results of all patients were comprehensively evaluated by two or more experienced interventional cardiovascular physicians. The appropriate intervention strategy was selected based on the angiography results and the patient's condition. Complete revascularization (CR) refers to anatomic CR, that is, lesions with a diameter of ≥2.0 mm and stenosis of ≥70% meet the criteria for successful interventional treatment (residual stenosis <30%). The patients who underwent revascularization during the first hospitalization and received additional planned revascularization within 1 month of the first revascularization were included in the analysis. This approach was defined as staged revascularization.

### 2.3 Endpoints and definitions

The endpoint of the study was MACE, which mainly included cardiovascular death, hospitalization for HF, and unplanned revascularization.

Hospitalization for heart failure was defined as hospitalization due to worsening heart failure requiring intravenous drug therapy. Unplanned revascularization was defined as either PCI or CABG for any reason.

### 2.4 Statistical analyses

Continuous data with normal distributions are expressed as means ± SD. Non-normally distributed variables are presented as medians with interquartile ranges (IQRs), which were analyzed using the Mann–Whitney *U* test. Categorical variables are presented as numbers and percentages, and comparisons were performed using the chi-square test.

Least absolute shrinkage and selection operator (LASSO–Cox regression and univariate Cox regression were used to identify the factors related to MACE. A nomogram prediction model for MACE in patients with IHF after PCI was developed based on Cox regression, including common influencing factors identified by univariate Cox regression and LASSO regression. The predictive performance of the model was evaluated using the concordance index (C-index) and the area under the curve (AUC) from receiver operating characteristic (ROC) analysis. Calibration curves were derived from Cox regression analysis using bootstrap methods to assess the consistency of the model. Decision curve analysis (DCA) was performed to evaluate the clinical utility of the nomogram. The Kaplan–Meier (K–M) method was used to compare the incidence of MACE between high-risk and low-risk patients as stratified by the model. Multivariate Cox regression was employed to identify independent factors associated with MACE. Statistical analyses were performed using SPSS version 29.0 and R software (version 4.4.1), with a *P*-value < 0.05 considered statistically significant.

## 3 Results

A total of 557 patients with IHF were treated at the main campus of the Second Hospital of Hebei Medical University between January 2019 and September 2023. Of these, 221 patients underwent PCI, 182 underwent coronary artery bypass grafting CABG, and 154 received OMT.

The median follow-up duration was 29.2 months, ranging from 0.70 to 66.6 months, with the last follow-up date on 25 July 2024. Among the 221 patients who underwent PCI, 9 were lost to follow-up, leaving 212 patients for analysis. Among them, 48 were in the MACE group, and 164 were in the non-MACE group. In the MACE group, there were 14 cardiovascular deaths, 16 hospitalizations for heart failure, 5 patients who underwent revascularization due to acute myocardial infarction, and 13 patients who underwent revascularization due to unstable angina.

### 3.1 Comparison of baseline data between the two groups

The baseline characteristics are shown in Table 1. Compared with the MACE group, the non-MACE group was younger (60.65 ± 10.94 vs. 65.60 ± 9.56 years, *P* = 0.005). In the non-MACE group, the use of angiotensin receptor–neprilysin inhibitors (ARNI) [124 (75.6%) vs. 21 (43.8%), *P* < 0.001] and sodium–glucose co-transporter-2 inhibitors (SGLT2i) [70 (42.7%) vs. 9 (18.8%), *P* = 0.030] was significantly higher, while the use of angiotensin-converting enzyme inhibitors (ACEI) or angiotensin receptor blockers (ARB) was lower [30 (18.3%) vs. 17 (35.4%), *P* = 0.012]. In addition, the number of residual diseased coronary arteries ≥2 was higher in the non-MACE group [35 (21.3%) vs. 19 (39.6%), *P* = 0.011].

**Table 1:** Comparison of baseline data between the two groups.

Clinical features	MACE (*n* = 48)	Non-MACE (*n* = 164)	*P*-value
Male [*n* (%)]	39 (81.3)	131 (79.9)	0.834
Age (*x̅* ± SD, years)	65.60 ± 9.56	60.65 ± 10.94	0.005
Body mass index (kg/m^2^)	25.25 ± 3.58	26.27 ± 3.69	0.090
Systolic pressure (mmHg)	131.23 ± 23.77	128.45 ± 20.43	0.426
Diastolic pressure (mmHg)	81.38 ± 14.76	80.07 ± 12.60	0.273
Heart rate (*x̅* ± SD)	73.02 ± 14.98	75.74 ± 13.70	0.237
Smoking [*n* (%)]	19 (39.6)	64 (39.0)	0.944
Drinking [*n* (%)]	14 (29.2)	43 (26.2)	0.685
Hypertension [*n* (%)]	30 (62.5)	103 (62.8)	0.969
Diabetes mellitus [*n* (%)]	18 (37.5)	72 (43.9)	0.430
Prior myocardial infarction [*n* (%)]	35 (72.9)	110 (67.1)	0.444
Prior PCI [*n* (%)]	22 (45.8)	66 (40.2)	0.489
NYHA classification III or IV	32 (66.7)	90 (54.9)	0.146
Aspirin [*n* (%)]	46 (95.8)	161 (98.2)	0.691
P2Y12 inhibitor [*n* (%)]	48 (100.0)	164 (100.0)	1.000
Statin [*n* (%)]	42 (97.7)	53 (100.0)	0.916
Beta-blocker [*n* (%)]	45 (93.8)	150 (91.5)	0.833
ARNI [*n* (%)]	21 (43.8)	124 (75.6)	<0.001
ACEI/ARB [*n* (%)]	17 (35.4)	30 (18.3)	0.012
SGLT2i [*n* (%)]	9 (18.8)	70 (42.7)	0.030
MRA [*n* (%)]	34 (70.8)	120 (73.2)	0.749
Hb (*x̅* ± SD, g/L)	139.25 ± 17.47	137.45 ± 16.89	0.520
ALB (*x̅* ± SD, g/L)	40.79 ± 4.41	41.21 ± 4.00	0.535
Scr [*M* (*P*_25_, *P*_75_), µmol/L]	85.00 (74.25, 109.75)	82.00 (71.00, 98.00)	0.156
LDL-C [*M*(*P*_25_, *P*_75_), mmol/L]	2.12 (1.73, 2.49)	2.13 (1.65, 2.73)	0.664
Lp(a) [*M*(*P*_25_, *P*_75_), mg/dL]	15.76 (6.37, 44.21)	13.05 (6.24, 35.68)	0.746
HbA1c [*M*(*P*_25_, *P*_75_), %]	6.20 (5.90, 7.00)	6.45 (5.80, 7.58)	0.445
Diseased coronary vessels			
LM [*n* (%)]	7 (14.6)	13 (7.9)	0.268
LAD [*n* (%)]	36 (75.0)	126 (76.8)	0.793
LCX [*n* (%)]	29 (60.4)	92 (56.1)	0.595
RCA [*n* (%)]	33 (68.8)	101 (61.6)	0.365
Two or three vessels disease [*n* (%)]	36 (75.0)	112 (68.3)	0.373
Complete revascularization [*n* (%)]	15 (31.3)	60 (36.6)	0.497
Chronic total occlusion [*n* (%)]	8 (16.7)	42 (25.6)	0.199
Total number of stents [*M* (*P*_25_, *P*_75_), *n*]	1 (1, 2)	2 (1, 2)	0.109
Total stent length [*M* (*P*_25_, *P*_75_), mm]	34.50 (24.00, 61.00)	39.50 (26.00, 61.00)	0.484
number of residual diseased CA ≥ 2 [*n* (%)]	19 (39.6)	35 (21.3)	0.011
LVEF [*M* (*P*_25_, *P*_75_), %]	39.05 (32.65, 44.58)	42.85 (37.20, 46.43)	0.013
LAD [*M* (*P*_25_, *P*_75_), mm]	39.00 (37.00, 43.00)	39.00 (36.00, 43.00)	0.489
LVEDD [*M* (*P*_25_, *P*_75_), mm]	61.00 (56.00, 68.00)	58.00 (53.00, 61.00)	0.002
IVS [*M* (*P*_25_, *P*_75_), mm]	10.00 (9.00, 11.00)	10.00 (9.00, 11.00)	0.506
*E*/*e*′[*M* (*P*_25_, *P*_75_)]	14.84 (10.62, 18.88)	13.92 (11.00, 18.35)	0.509
Moderate or severe MR [*n* (%)]	21 (43.8)	46 (28.0)	0.040
Ventricular aneurysm [*n* (%)]	8 (16.7)	27 (16.5)	0.973

Data presented as mean ± SD, *n* (%), median (IQR). PCI, percutaneous coronary intervention; NYHA, New York Heart Association; ARNI, angiotensin receptor–neprilysin inhibitor; ACEI, angiotensin-coverting enzyme inhibitors; ARB, angiotensin receptor blocker; SGLT2i, sodium glucose co-transporter 2 inhibitors; MRA, mineralocorticoid receptor antagonist; Hb, hemoglobin; ALB, serum albumin; Scr, serum creatinine; LDL-C, low-density lipoprotein cholesterol; Lp(a), serum lipoprotein(a); HbAlc, Hemoglobin A1C; LM, left main artery; LAD, left anterior descending artery; LCX, left circumflex artery; RCA, right coronary artery; CA, coronary arteries; LVEF, left ventricular ejection fraction; LAD, left atrial dimension; LVEDD, left ventricular end-diastolic dimension; IVS, interventricular septal; *E*/*e*′, early diastolic transmitral velocity to early diastolic mitral annular velocity; MR, mitral regurgitation.

Regarding echocardiographic parameters, the non-MACE group had lower baseline LVEF at admission [39.05 (32.65, 44.58) vs. 42.85 (37.20, 46.43), *P* = 0.013], larger left ventricular end-diastolic dimension (LVEDD) [61.00 (56.00, 68.00) vs. 58.00 (53.00, 61.00), *P* = 0.002], and a higher proportion of moderate to severe mitral regurgitation (MR) [46 (28.0%) vs. 21 (43.8%), *P* = 0.040].

### 3.2 Screen the influencing factors

The results of univariate Cox regression and LASSO regression are shown in Table 2. All the factors from baseline data were included in univariate Cox regression analysis, which identified age (HR = 1.030, 95% CI: 1.001–1.061, *P* = 0.043), NYHA classification III or IV (HR = 1.998, 95% CI: 1.093–3.654, *P* = 0.025), number of residual diseased coronary arteries ≥2 (HR = 1.885, 95% CI: 1.055–3.370, *P* = 0.032), LVEF (HR = 0.944, 95% CI: 0.905–0.985, *P* = 0.008), LVEDD (HR = 1.051, 95% CI: 1.018–1.084, *P* = 0.002), and moderate and severe MR (HR = 1.793, 95% CI:1.012–3.177, *P* = 0.045) as risk factors for MACE. In addition, the use of ARNI during follow-up (HR = 0.466, 95% CI: 0.261–0.835, *P* = 0.010) was identified as a protective factor.

**Table 2:** Results of univariate Cox regression and LASSO regression.

Clinical features	Univariate Cox regression			LASSO regression	
Independent variable	*OR*	*95% CI*	*P*	Independent variable	Coeff min lambda
Age^a^	1.030	1.001–1.061	0.043	Age^a^	0.0039
NYHA classification III or IV^a^	1.998	1.093–3.654	0.025	NYHA classification III or IV^a^	0.2026
Residual diseased CA ≥ 2^a^	1.885	1.055–3.370	0.032	Residual diseased CA ≥ 2^a^	0.1805
LVEF^a^	0.944	0.905–0.985	0.008	LVEF^a^	−0.0016
LVEDD^a^	1.051	1.018–1.084	0.002	LVEDD^a^	0.0257
Moderate or severe MR	1.793	1.012–3.177	0.045		
ARNI^a^	0.466	0.261–0.835	0.010	ARNI^a^	−0.2672
ACEI/ARB	1.277	0.689–2.368	0.438		
SGLT2i	0.524	0.250–1.099	0.087		
				E/e’	−0.0020

NYHA, New York Heart Association; CA, coronary artery; LVEF, left ventricular ejection fraction; LVEDD, left ventricular end-diastolic dimension; MR, mitral regurgitation; ARNI, angiotensin receptor–neprilysin inhibitor; ACEI, angiotensin-converting enzyme inhibitor; ARB, angiotensin receptor blocker; SGLT2i, sodium glucose co-transporter 2 inhibitors.

^a^ Common influencing factors screened by univariate Cox regression and LASSO regression.

**Figure 1:**
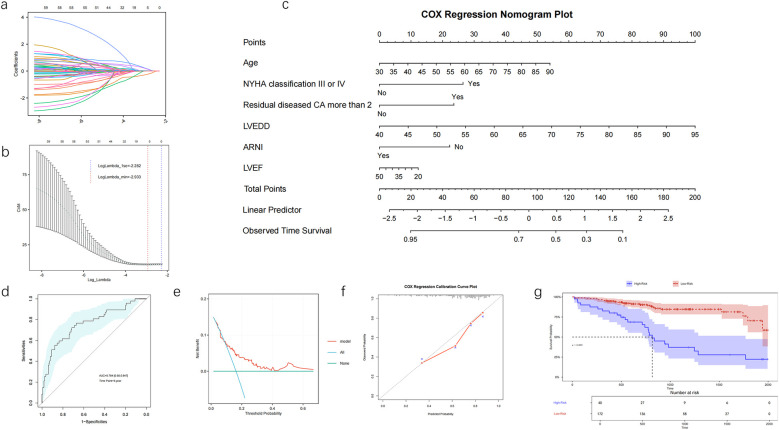
The development and evaluation of a nomogram prediction model in patients with IHF after PCI, LASSO regression coefficient profiles **(a)** and 10-fold cross-validation **(b)** nomogram for long-term prognosis in patients with IHF after PCI **(c)**. ROC curve of the nomogram model **(d)**, decision curve analysis of the nomogram **(e)**, calibration curve for the nomogram by bootstrap with 1,000 repetitions **(f)**. Kaplan-Meier survival curves of No-MACE survival for patients with high and low risk stratified by the model **(g)**. NYHA, New York Heart Association; CA, coronary artery; LVEF, left ventricular ejection fraction; LVEDD, left ventricular end-diastolic dimension; ARNI, angiotensin receptor–neprilysin inhibitor.

The results of the LASSO regression analysis showed that age, NYHA classification III or IV, number of residual diseased coronary arteries ≥2, LVEF, LVEDD, early diastolic transmitral velocity to early diastolic mitral annular velocity (*E*/*e*′), and the use of ARNI during follow-up were significantly associated with MACE based on the *λ* criterion at minimum values (Figures 1a,b).

### 3.3 Development and evaluation of a nomogram prediction model

A nomogram prediction model was developed based on common influencing factors identified by Cox regression and LASSO regression, including age, NYHA classification III or IV, number of residual diseased coronary arteries ≥2, LVEF, LVEDD, and the application of ARNI during follow-up (Figure 1c). The AUC for the 5-year ROC analysis of the model was 0.764 (95% CI: 0.680–0.847), and the concordance index (C-index) was 0.713, indicating that the model has good discriminatory ability at the 5-year mark (Figure 1d). The calibration curve and DCA demonstrated that the model had good consistency and clinical utility. The DCA further indicated that when the threshold probability ranges from 10% to 65%, the application of the model yields greater benefits, especially in the range of 15% to 60%, suggesting that the model can effectively guide treatment decisions (Figure 1e). The calibration curve showed excellent concordance between the model's predictions and actual observations (Figure 1f). Using this model, we stratified patients into high-risk and low-risk groups. The K–M analysis revealed that the high-risk group had a significantly higher incidence of MACE compared with the low-risk group (Figure 1g).

### 3.4 Nomogram validation

A total of 91 patients with IHF treated at the branch campus of the Second Hospital of Hebei Medical University between January 2019 and September 2023 were included in the external validation dataset. The baseline characteristics between patients from the two campuses are shown in Supplementary Table S1.

**Figure 2:**
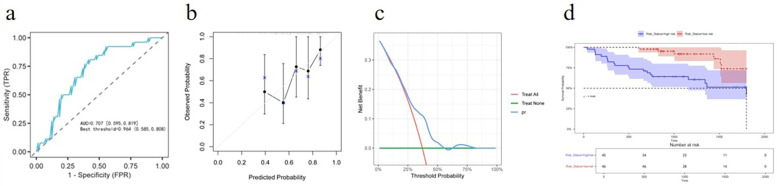
External validation for predictive accuracy of the nomogram. **(a)** ROC curves for the external validation dataset. **(b)** Calibration curves for the external validation dataset. **(c)** Decision curve analysis for the external validation dataset. **(d)** Kaplan–Meier survival curves of non-MACE survival for patients with high and low risk stratified by the model in the external validation dataset.

The nomogram maintained a good predictive accuracy with an AUC of 0.707 (95% CI: 0.595–0.819) (Figure 2a), and the C-index was 0.691. The calibration curve also had a good performance (Figure 2b), and the DCA indicated that patients achieved a high net benefit for the predicted probability thresholds between 20% and 50% (Figure 2c). The K–M analysis revealed that the high-risk group had a significantly higher incidence of MACE compared with that of the low-risk group (Figure 2d).

**Figure 3:**
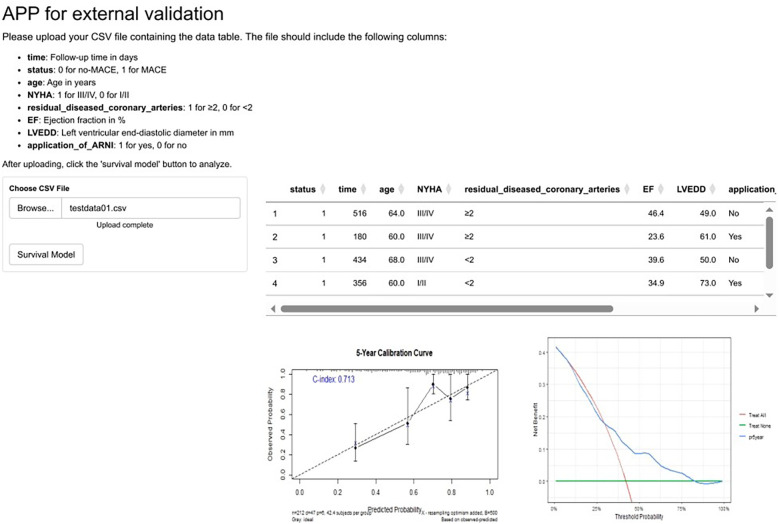
An online app for external validation of the prediction model. To enable researchers from diverse institutions to perform external validation of our model using their own datasets, we have developed an application available at https://hebmuguan01.shinyapps.io/APPSU/. By following the provided guidelines, users can upload their data, generate calibration and DCA curves, and comprehensively assess the model's performance in external validation cohorts. The figure demonstrates the app's functionality using the hypothetical “testdata.csv” as an example. MACE, major adverse cardiovascular events; NYHA, New York Heart Association; LVEF, left ventricular ejection fraction; LVEDD, left ventricular end-diastolic dimension; ARNI, angiotensin receptor–neprilysin inhibitor.

Moreover, to achieve broader model validation, we developed a web-based application and deployed it at https://hebmuguan01.shinyapps.io/APPSU/. Researchers from other institutions can use their own datasets as external validation sets by following the instructions on the webpage—uploading “.csv” files and clicking the corresponding buttons to complete external validation. The system automatically generates calibration curves and DCA curves (Figure 3).

### 3.5 Results of multivariate Cox regression

**Figure 4:**
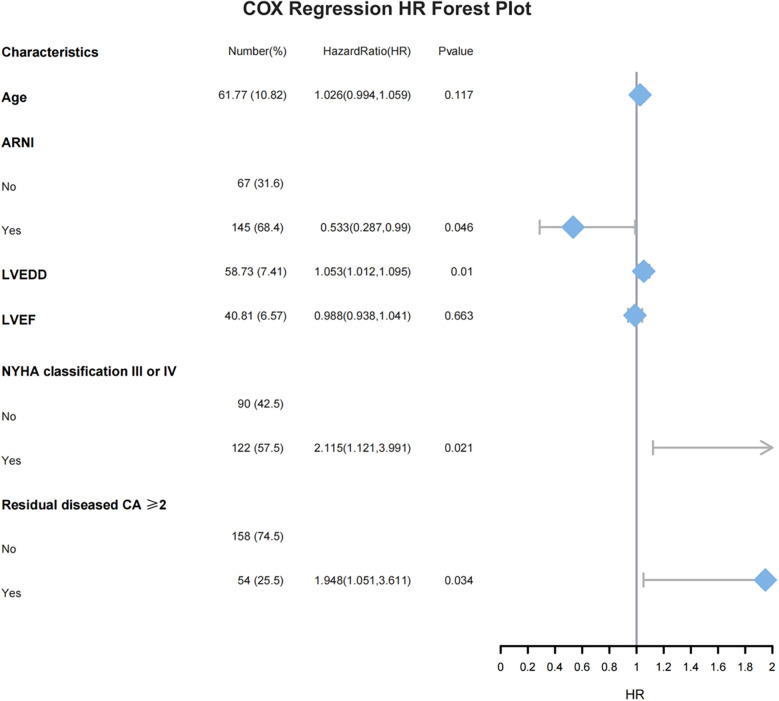
Multivariate Cox regression HR forest plot, ARNI, angiotensin receptor–neprilysin inhibitor; LVEDD, left ventricular end-diastolic dimension; LVEF, left ventricular ejection fraction; NYHA, New York Heart Association; CA, coronary artery.

The common influencing factors identified by Cox regression and LASSO regression, including age, NYHA classification III or IV, number of residual diseased coronary arteries ≥2, LVEF, LVEDD, and the application of ARNI during follow-up, were included in multivariate Cox regression. The results showed that NYHA classification III or IV (HR = 2.115, 95% CI: 1.121–3.991, *P* = 0.021), number of residual diseased coronary arteries ≥2 (HR = 1.984, 95% CI: 1.051–3.611, *P* = 0.034), and LVEDD (HR = 1.053, 95% CI: 1.012–1.095, *P* = 0.01) were independent risk factors for MACE. In contrast, the use of ARNI during follow-up (HR = 0.533, 95% CI: 0.287–0.99, *P* = 0.046) was an independent protective factor for MACE (Figure 4).

## 4 Discussion

In this retrospective analysis, univariate Cox regression and LASSO regression all showed that age, NYHA classification III or IV, residual diseased coronary arteries ≥2, LVEF, LVEDD, and the application of ARNI during follow-up were significantly correlated with the MACE. A nomogram prediction model based on the above six factors has a good differentiation ability. The calibration curve and the DCA indicated that the model has good consistency and clinical application value. Furthermore, the nomogram was validated externally using data from the branch campus of the hospital to confirm its reliability. Multivariate Cox regression showed that NYHA classification III or IV, residual diseased coronary arteries ≥2, and LVEDD were independent risk factors for MACE, and ARNI was used during follow-up as an independent protective factor for MACE. These findings can assist clinicians in identifying patients at high risk of poor prognosis, improving prognosis assessment, and providing a reference for developing clinical management strategies.

Current therapies can significantly improve the clinical symptoms and quality of life in patients with HF. However, compared with non-ischemic HF, patients with IHF have a higher incidence of adverse events and worse prognosis (12–14). Moreover, studies have shown that IHF is an independent predictor of long-term mortality and hospital readmission (13, 14). In this study, we conducted a long-term follow-up of patients with IHF after PCI, with an average follow-up of 29.2 months, and observed a 29% incidence of MACE, which was lower than reported in previous studies 11 (15). This may be due to the relatively short follow-up time in our study, the younger age of the included patients, and differences in the definition of MACE. In addition, advances in stent technology and pharmacotherapy for patients with IHF in recent years may also account for the lower event rate in this study.

The NYHA classification is a simple risk stratification tool that not only provides a rapid assessment of cardiac function in patients with HF but also has strong prognostic value (16). However, the NYHA classification is a subjective evaluation index, and when patients’ symptoms are not pronounced, the assessment results are more dependent on subjective perceptions, making it difficult to distinguish between grade I and grade II patients (17). As a result, it has better predictive value for patients with NYHA class III–IV. The findings of this study are consistent with previous research, where NYHA class III–IV was identified as an independent risk factor for poor prognosis in patients with HF (18, 19).

Myocardial infarction can lead to ventricular remodeling, which is characterized by structural and functional changes in the heart, such as increased chamber volume, thinning of the ventricular wall, and reduced LVEF. In patients with IHF, coronary artery lesions are often diffuse and complex, causing long-term myocardial ischemia, which results in the necrosis of cardiomyocytes and their gradual replacement by fibrous tissue. Eventually, the ventricular wall thins, and the ventricular cavity gradually expands. Therefore, LVEDD is an important diagnostic index of ventricular remodeling (20–22). The results of this study suggest that increased LVEDD is an independent risk factor for long-term poor prognosis in patients with IHF after PCI. The larger the baseline LVEDD, the more severe the left ventricular remodeling. Poor ventricular remodeling reduces myocardial contractility, increases the risk of HF, and lowers survival rates (23).

Activation of the renin–angiotensin–aldosterone system (RAAS) is one of the important mechanisms for ventricular remodeling (20), and the activation level is closely related to the severity and adverse outcomes of patients with HF (24). ARNI is the first angiotensin receptor–neprilysin inhibitor, which not only targets the natriuretic peptide system but also inhibits the RAAS, effectively improving ventricular remodeling, dilating blood vessels, and reducing sympathetic nervous system activity. Numerous studies have confirmed that the use of ARNI in HF patients not only lowers N-terminal pro-B-type natriuretic peptide levels and increases LVEF but also outperforms ACEI and ARB in improving cardiac function and reversing ventricular remodeling (25–27). In addition, ARNI reduces the risk of cardiovascular death, heart failure hospitalizations, and the progression of IHF in outpatient settings (28–31). The results of this study demonstrate that the use of ARNI is an independent protective factor in reducing the occurrence of long-term MACE in patients with IHF. This suggests that in the clinical management of patients with IHF, clinicians should prioritize not only revascularization but also optimal medical therapy, including ARNI.

CR has long been the goal of coronary artery revascularization. A large body of evidence has confirmed that incomplete revascularization (ICR) negatively affects long-term prognosis and increases the risk of adverse cardiovascular events (32–34). The residual Synergy between PCI with Taxus and Cardiac Surgery (SYNTAX) score, a quantitative tool for assessing the degree of ICR, has been shown to be a predictor of clinical outcomes in patients post-PCI (35–37). However, the residual SYNTAX score has several limitations, such as complexity in calculation, low repeatability, improper weight allocation of coronary lesions, and failure to consider clinical characteristics. In this study, a simpler method was used: residual diseased coronary arteries were defined as vessels that met the criteria for revascularization but did not receive interventional treatment. The results showed that having two or more residual diseased coronary arteries was an independent risk factor for long-term MACE in patients with IHF after PCI, highlighting the adverse impact of residual coronary artery disease on long-term prognosis.

It is important to note that coronary artery lesions in IHF patients are often complex, with a high prevalence of multivessel disease and chronic occlusive lesions, which can prolong PCI procedures, increase radiation exposure, and raise the dose of contrast agents used. In addition, IHF patients frequently have more comorbidities and poor heart function, which increases the risk of PCI complications. Therefore, a comprehensive evaluation of a patient's clinical condition is crucial, rather than blindly pursuing complete anatomical CR. Functional assessment should be used to accurately identify the coronary arteries that require complete revascularization, reducing the risk of unnecessary PCI. Thus, “rational” ICR may represent a better revascularization strategy (38).

This study had several limitations. First, it was a single-center, retrospective study with a small sample size, which inherently introduces selection bias and data absence. Second, due to the complexity and poor reproducibility of the SYNTAX score, baseline SYNTAX and residual SYNTAX scores were not used to evaluate coronary artery complexity and the completeness of revascularization, potentially affecting revascularization completeness assessment. Third, the lack of comparison of clinical outcomes with those of patients receiving medical therapy or CABG may restrict broader conclusions. In future work, we will seek to validate these findings in larger, multicenter cohorts to enhance their generalizability. Furthermore, we should conduct direct comparative analyses to evaluate the model's performance across different treatment strategies, which is essential to define its clinical utility.

## 5 Conclusions

In this study, the occurrence of MACE after PCI in patients with IHF was evaluated as the clinical outcome, and the factors influencing long-term MACE were analyzed. The nomogram prediction model, based on age, NYHA classification III or IV, residual diseased coronary arteries ≥2, LVEF, LVEDD, and the use of ARNI during follow-up, demonstrated high predictive value for long-term MACE in patients with IHF after PCI. NYHA classification III or IV, residual diseased coronary arteries ≥2, and LVEDD were identified as independent risk factors for MACE, while the use of ARNI during follow-up was found to be an independent protective factor.

## Data Availability

The original contributions presented in the study are included in the article/Supplementary Material, further inquiries can be directed to the corresponding author.
