# Supplementary material for: Analysis of risk factors and development of a prediction model for long-term prognosis in patients with ischemic heart failure after percutaneous coronary intervention
Source: Front Cardiovasc Med. 2025 Oct 27;12:1545079. doi: 10.3389/fcvm.2025.1545079 (PMC12598039; doi:10.3389/fcvm.2025.1545079)
Supplement: Supplementary file 1 [file Table1.docx]

**Table S1 Comparison of baseline characteristics between patients from the two campuses**

|  | main campus  (*n*=212) | branch campus  (n=91) | *P*值 |
| --- | --- | --- | --- |
| Male[n（%）] | 170 (80.2) | 78 (85.7) | 0.253 |
| Age(x̅±SD，years) | 61.77±10.82 | 60.86±11.40 | 0.509 |
| Body mass index（Kg/m^2^） | 26.04±3.68 | 26.07±4.25 | 0.941 |
| Systolic pressure（mmHg） | 129.08±21.20 | 124.81±19.67 | 0.190 |
| Diastolic pressure（mmHg） | 80.37±13.09 | 78.27±11.80 | 0.102 |
| Heart rate (x̅±SD) | 75.13±14.01 | 75.75±13.85 | 0.723 |
| Smoking [n（%）] | 83 (39.2) | 42 (46.2) | 0.256 |
| Drinking [n（%）] | 57 (26.9) | 33 (33.0) | 0.284 |
| Hypertension [n（%）] | 133 (62.7) | 51 (56.0) | 0.274 |
| Diabetes mellitus [n（%）] | 90 (42.5) | 34 (37.4) | 0.409 |
| Prior myocardial infarction [n（%）] | 145 (68.4) | 52 (57.1) | 0.060 |
| Prior PCI [n（%）] | 88 (41.5) | 31 (34.1) | 0.224 |
| NYHA classification III or IV | 122 (57.5) | 43 (47.3) | 0.099 |
| Aspirin [n（%）] | 190 (89.6) | 85 (93.4) | 0.297 |
| P2Y12 inhibitor [n（%）] | 212 (100.0) | 91 (100.0) | 1.000 |
| Statin [n（%）] | 207 (97.6) | 89 (97.8) | 0.932 |
| Beta-blocker [n（%）] | 195 (92.0) | 83 (91.2) | 0.823 |
| ARNI[n（%）] | 145 (68.4) | 54 (59.3) | 0.128 |
| ACEI/ARB [n（%）] | 47 (22.2) | 23 (25.3) | 0.557 |
| SGLT2i [n（%）] | 86 (40.6) | 40 (44.0) | 0.583 |
| MRA [n（%）] | 154 (70.6) | 64 (70.3) | 0.681 |
| Hb(x̅±SD，g/L) | 137.86±17.00 | 140.97±14.97 | 0.132 |
| ALB(x̅±SD，g/L) | 41.11±4.09 | 40.90±4.19 | 0.676 |
| Scr[*M*(*P*_25_，*P*_75_)，umol/L] | 82.00 (72.00, 100.75) | 79.80 (73.20, 94.00) | 0.269 |
| LDL-C[*M*(*P*_25_，*P*_75_)，mmol/L] | 2.13 (1.67, 2.67) | 2.19 (1.79, 2.60) | 0.473 |
| Lp(a)[*M*(*P*_25_，*P*_75_)，mg/dl] | 13.67 (6.24, 36.45) | 16.70 (7.95, 50.00) | 0.164 |
| HbA1c[*M*(*P*_25_，*P*_75_)，%] | 6.40 (5.80, 7.50) | 6.00 (5.40, 7.30) | 0.015 |
| Diseased coronary vessels |  |  |  |
| LM[n（%）] | 20 (9.4) | 7 (7.7) | 0.626 |
| LAD[n（%）] | 162 (76.4) | 63 (69.2) | 0.190 |
| LCX[n（%）] | 121 (57.1) | 57 (62.6) | 0.367 |
| RCA[n（%）] | 134 (63.2) | 56 (61.5) | 0.783 |
| Two or Three Vessels disease [n（%）] | 148 (69.8) | 57 (62.6) | 0.221 |
| Complete revascularization[n（%）] | 75 (35.4) | 41 (48.1) | 0.112 |
| Chronic total occlusion[n（%）] | 50 (23.6) | 27 (29.7) | 0.265 |
| Total number of stents [*M*(*P*_25_，*P*_75_)，n] | 1 (1, 2) | 2 (1, 2) | 0.931 |
| Total stent length[*M*(*P*_25_，*P*_75_)，mm] | 37.50 (26.00, 61.00) | 33.00 (20.00,62.00) | 0.386 |
| number of residual diseased CA≥2  [n（%）] | 54 (25.5) | 26 (28.6) | 0.575 |
| LVEF[*M*(*P*_25_，*P*_75_)，%] | 42.25 (35.70, 46.18) | 41.30 (37.00,46.44) | 0.657 |
| LAD[*M*(*P*_25_，*P*_75_)，mm] | 39.00 (36.00, 43.00) | 39.00 (36.00, 42.00) | 0.284 |
| LVEDD[*M*(*P*_25_，*P*_75_)，mm] | 58.00 (54.00, 62.75) | 58.00 (54.00, 62.00) | 0.523 |
| IVS[*M*(*P*_25_，*P*_75_)，mm] | 10.00 (9.00, 11.00) | 9.00 (8.00, 10.00) | ＜0.001 |
| E/e’[*M*(*P*_25_，*P*_75_)] | 14.00 (10.93, 18.50) | 13.80 (10.00, 18.00) | 0.456 |
| Moderate or severe MR [n（%）] | 61 (31.6) | 32 (35.2) | 0.545 |
| ventricular aneurysm[n（%）] | 35 (16.5) | 10 (11.0) | 0.215 |

Data presented as mean ± SD, n (%), median (IQR). Abbreviations: PCI: percutaneous coronary intervention; NYHA: New York Heart Association; ARNI: angiotensin receptor-neprilysin inhibitor; ACEI: angiotensin coverting enzyme inhibitors; ARB: angiotensin receptor blocker; SGLT2i: sodium glucose co-transporter 2 inhibitors; MRA: mineralocorticoid receptor antagonist; Hb: hemoglobin; ALB: serum albumin; Scr: serum creatinine; LDL-C: low-density lipoprotein-cholesterol; Lp(a): serum lipoprotein(a); HbAlc: Hemoglobin A1C; LM: left main artery; LAD: left anterior descending artery; LCX: left circumflex artery; RCA: right coronary artery; CA: coronary arteries; LVEF: left ventricular ejection fraction; LAD: left atrial dimension; LVEDD: left ventricular end-diastolic dimension; IVS: inter-ventricular septal; E/e’:early diastolic transmitral velocity to early diastolic mitral annular velocity; MR: mitral regurgitation.
